# Editorial: Food-Based Dietary Guidelines: The Relevance of Nutrient Density and a Healthy Diet Score

**DOI:** 10.3389/fnut.2020.576144

**Published:** 2020-12-14

**Authors:** Ellen G. H. M. van den Heuvel, Lisette C. P. G. M. de Groot, Inge Tetens, Edith Feskens, Monique M. Raats, Jan Steijns

**Affiliations:** ^1^FrieslandCampina, Amersfoort, Netherlands; ^2^Division of Human Nutrition and Health, Wageningen University and Research, Wageningen, Netherlands; ^3^Department of Nutrition, Exercise and Sports, Vitality - Centre for Good Older Lives, University of Copenhagen, Copenhagen, Denmark; ^4^Food Consumer Behaviour and Health Research Centre, Faculty of Health and Medical Sciences, University of Surrey, Guildford, United Kingdom

**Keywords:** nutrient rich food score, dietary muscle health score, bioavailability, drivers of consumption, protein, vitamin B12, vitamin K, dairy

Over time the main drivers of eating have shifted from ensuring adequate caloric intake to maintaining optimal health and socializing with appetizing and desirable foods. Sustainable food production is also now considered important. The recent EAT-Lancet Commission (1) suggested a change in the current diet to include lower amounts of animal source foods and a high diversity of plant-based foods, to meet both nutrient requirement and sustainability criteria. The key aim of this Research Topic is to provide a picture of the relevance of different measures of nutritional quality in relation to healthiness of diets such as proposed by the EAT-Lancet with focus on major food- and target groups, whilst taking drivers of food consumption into account.

Nutrients work together, rather than in isolation, and potential beneficial effects of specific nutrients will only appear if intake of other nutrients is optimal. To separate foods that are energy-dense from those that are nutrient-rich, Drewnoski (2) developed a score to assess nutrient density of foods. The nutrient rich food (NRF) score X.Y is based on recommended daily allowances for adult populations with X representing nutrients to encourage and Y nutrients to limit. Within this Research Topic this score was adapted specifically for the European older population, as their proportion is rising and consequently the burden of degenerative diseases (Berendsen et al.) The Elderly NRF score (E-NRF7.3) was composed of nutrients, shown to be of inadequate intake in the aging population, defined as nutrients of public health relevance, and associated with relevant health outcomes. Vegetables, bread, potatoes, and milk and products contributed the most to the new E-NRF7.3 score, which also correlated with a greater adherence to a healthful diet (Berendsen et al.) Moreover, the higher the score the higher the serum folate levels in both Dutch and Polish elderly, the lower the homocysteine (the Netherlands only), and the higher the vitamin B12 levels were (Poland only) (Kramer et al.) The above mentioned articles confirm available literature that the nutrient density score can distinguish nutritious basic food product from unhealthy alternatives, and therewith show validity of the score. However, in the nutrient density score discrimination between healthy and unhealthy foods could still be improved. Recently. Drewnowsky et al., designed an hybrid nutrient density score, which also includes food groups. He showed that this score would also include the healthfulness of foods not readily captured by a score based on nutrients only (3).

Decreasing consumption of animal foods may increase the risk of certain nutrient deficiencies, such as vitamin B12. An overview of vitamin B12 intake and status in different target groups showed that specifically dairy consumption seemed to be a stronger determinant of vitamin B12 status (Obeid et al.) Cheese is a source of vitamin K_2_, a micronutrient with demonstrated positive results on cardiovascular-related outcomes. However, cheese did not add extra to the association between a healthy diet score and incident T2DM or all-cause mortality (Dekker et al.) Nutrient dense animal food products rich in vitamin B12 or vitamin K, are also rich in other nutrients such as calcium, iron, and proteins. A study showed that eliminating dairy foods and replacing them iso-calorically by alternatives with more unsaturated fats led to deficits in some key nutrients and to lower NRF9.3 scores (Rehm and Drewnowski) These papers highlight the fact that the nutrient-based dietary guidance proposed by the EAT-Lancet commission may not account for the complexity of dietary patterns and may have unintended unfavorable nutritional consequences.

In addition to a diversity of single nutrients, a healthy eating pattern should according to Burd et al. include the ingestion of high quality protein, preferably in adequate amounts across all meals throughout the day. Given its role in muscle function and metabolic health, protein recommendations should be adapted to reflect needs throughout life/health-stage, acknowledging food matrix interactions and potential food structure changes resulting from heat treatment, while also ensuring other nutrient adequacies. Schacht et al. therefore developed an older adult-specific dietary muscle health score that is based on associations between food groups and muscle function outcomes. The advantage of this score being it includes nutrient-density, the well-known interactions between nutrients and the bioavailability of these nutrients from the matrix, which is further discussed by Melse-Boonstra.

Reducing the share of ultra-processed foods in the diet has been suggested by Gupta et al. to be a means of improving diets, albeit at a higher cost. They showed that ultra-processed foods tend to be low-cost and represent energy-dense, nutrient poor foods while unprocessed meat, poultry, fish, low fat milk, vegetables, and fruit had lower energy density, higher NRF9.3 nutrient density scores and were considerably more expensive. Drewnowski and Richonnet explored whether the main ingredient in a food could be used as a marker for diet quality. With a focus on snacks, they found that dairy and fruit, when listed as first ingredients, were associated with higher NRF scores. Therefore, the main ingredients in a food have the potential to be used by consumers to quickly differentiate snacks on nutritional value. However, the question remains whether this potentially easy to understand information is sufficient to drive consumers to a NRF choice. Cost also appears to be an important driver of choice, as partly underscored by results of Maillot et al. who found that children from lower-income groups drank less low fat milk and water and more whole milk and caloric sugar sweetened beverages. Another important driver of consumption is food preference. Nutrient poor foods often differ in their taste profiles from NRF by having a sweet, salty, fatty taste profile. Liem and Russell concluded that there is large individual variation in adult consumers' preference for sweet taste, salt taste, and fat taste/texture. This may offer opportunities for other important drivers than taste e.g., smell and texture, to impact food choice and consumption and to improve the nutritional quality of diets.

In conclusion, the present Research Topic provides several examples that both plant- and animal foods are needed to ensure optimal diets in relation to optimal health. The present Research Topic also suggests that the ratio between plant- and animal products needs further research, in which the use of a scientifically valid NRF score might play a key role. Drivers to improve the consumer's intake of NRF are likely to include cost and easy to understand information, in combination with an optimal sensory profile.

## Author Contributions

All authors listed have made a substantial, direct and intellectual contribution to the work, and approved it for publication.

## Conflict of Interest

EH and JS are employees at FrieslandCampina, a dairy company. Any opinions, findings, conclusions, or recommendations expressed in the current work are those of the authors and do not necessarily reflect the views of FrieslandCampina. The remaining authors declare that the research was conducted in the absence of any commercial or financial relationships that could be construed as a potential conflict of interest.
